# Identification of *LTBP2* gene polymorphisms and their association with thoracolumbar vertebrae number, body size, and carcass traits in Dezhou donkeys

**DOI:** 10.3389/fgene.2022.969959

**Published:** 2022-11-22

**Authors:** Ziwen Liu, Tianqi Wang, Xiaoyuan Shi, Xinrui Wang, Wei Ren, Bingjian Huang, Changfa Wang

**Affiliations:** Liao Cheng Research Institute of Donkey High-Efficiency Breeding, Liaocheng University, Liaocheng, China

**Keywords:** thoracolumbar vertebrae, Dezhou donkey, *LTBP2*, single nucleotide variants (SNVs), association analysis

## Abstract

The number of thoracolumbar vertebrae in Dezhou donkeys varies from 22 to 24 and is associated with body size and carcass traits. In mammals, the latent transforming growth factor beta binding protein 2 (*LTBP2*) has been found to have some functions in the development of thoracolumbar vertebrae. The relationship between *LTBP2* and TLN (the number of thoracolumbar vertebrae) of Dezhou donkeys is yet to be reported. The purposes of this study are as follows: 1) to quantify the effect of thoracolumbar vertebrae number variation of Dezhou donkeys on body size and carcass trait; 2) to study the distribution of single nucleotide variants (SNVs) in the *LTBP2* gene of Dezhou donkeys; and 3) to explore whether these SNVs can be used as candidate sites to study the mechanism of Dezhou donkey muti-thoracolumbar vertebrae development. The TLN, body size, and carcass traits of 392 individuals from a Dezhou donkey breed were recorded. All animals were sequenced for *LTBP2* using GBTS liquid chip and 16 SNVs were used for further analysis. We then analyzed the relationship between these SNVs with TLN, body size, and carcass traits. The results showed that: 1) c.5547 + 860 C > T, c.5251 + 281 A > C, c.3769 + 40 C > T, and c.2782 + 3975 A > G were complete genetic linkages and significantly associated with thoracic vertebrae number (TN) (*p* < 0.05) (wild-type homozygotes had more TN than heterozygotes); 2) c.1381 + 768 T > G and c.1381 + 763 G > T were significantly associated with lumber vertebrae number (LN) (*p* < 0.05); 3) c.1003 + 704 C > T, c.1003 + 651 C > T, c.1003 + 626 A > G, and c.812 + 22526 T > G were significantly associated with chest circumference (CHC), front carcass weight (CWF), after carcass weight (CWA), and carcass weight (CW) (*p* < 0.05) (wild-type homozygotes were larger than other genotypes in CHC, CWF, CWA, and CW); and 4) the effect of variation is not consistent in c.565 + 11921 A > G, c.565 + 6840 A > G, c.565 + 3453 C > T, and c.494 + 5808 C > T. These results provide useful information that the polymorphism of *LTBP2* is significantly associated with TLN, body size, and carcass traits in Dezhou donkeys, which can serve as a molecule marker to improve donkey production performance.

## 1 Introduction

In mammals, the vertebral column consists of cervical, thoracic, lumbar, sacral, and caudal vertebrae. The number of vertebrae, especially the thoracolumbar vertebrae number, is an important economic trait in domestic animals because it is related to the length of the carcass and the size of the body (Borchers et al., 2004). The number of thoracic vertebrae (TN) is equal to the number of ribs, which are among the most valuable parts of the meat in the China market, and the number of lumbar vertebrae (LN) is bound up with abdominal muscle. The variation in the number of thoracolumbar vertebrae (TLN) has been observed in many mammalian species, such as in sheep; there are 13 thoracic vertebrae and 6 lumbar vertebrae (T13L6) for the majority of sheep, while the carcass length of T13L7 and T14L6 increases by 2.22 cm and 2.93 cm, respectively, compared with normal T13L6, and carcass weight (CW) increases about 1.68 kg and 1.90 kg (Donaldson et al., 2013; Li et al., 2017). In pig breeds, modern Western pig breeds, such as Duroc, Large White, and Landrace, have more (*n* = 21–23) TLN than Chinese indigenous breeds (*n* = 19), generally (King and Roberts, 2010; Mikawa et al., 2011; Liu et al., 2020), and one extra vertebrae expands the carcass length by 80 mm in bacon pigs (King and Roberts, 2010).

The vertebral column originates from the pre-somatic mesoderm under regulation of the notochord, which means the number of vertebrae in a region may vary connaturally, and this has been accurately determined in the embryonic stage (Greene and Copp, 2009). The variability of the vertebral column may arise from cranial-caudal border shifts, which take place when there is a somatic shift from the typical distribution of vertebral segments in a region; this may be one of the reasons for the inconsistent TLN (Thawait et al., 2012). The multi-vertebrae number trait is complex and regulated by genetics; up to now, several genes have been proved to have the function of regulating the development of vertebrae. Two quantitative trait loci (QTL) have been identified to regulate the vertebrate development in pigs: one on chromosome 1 and another one on chromosome 7; through more in-depth research, the nuclear receptor subfamily 6, group A, member 1 (*NR6A1*) gene (c.748 Pro 192 Leu) and vertebrae development-associated (*VRTN*) gene (g.20311-20312 ins291) have been proved to be candidate genes affecting spinal development (Mikawa et al., 2005; Mikawa et al., 2007; Mikawa et al., 2011; Ren et al., 2012; Fan et al., 2013; Burgos et al., 2015; Zhang et al., 2016). Association analysis has revealed significant associations between the single nucleotide variants (SNVs) (rs414302710: A > G) in the exon-8 of the *NR6A1* gene with the number of lumbar vertebrae (Zhang et al., 2019). In addition to this, many genes have been found to be associated with the development of the vertebrae, such as the homeobox gene family (Rijli et al., 1995; Wellik, 2007; Gomez et al., 2008), *GDF11* (Li et al., 2010), and *Btg2* (Park et al., 2004), but most of these studies have focused on model animals, such as mice and fish.

**FIGURE 1:**
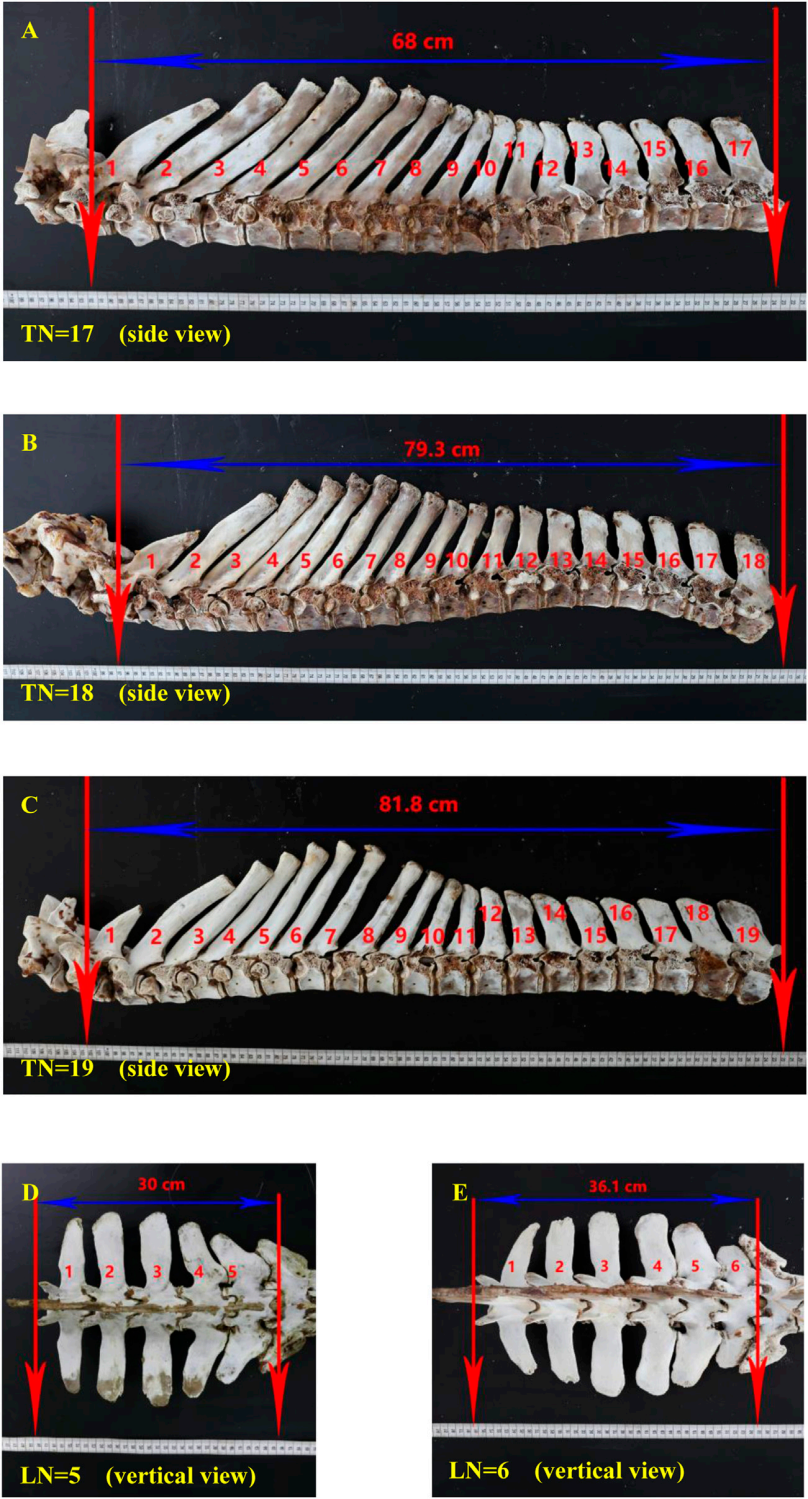
Thoracic and lumbar vertebrae specimens of Dezhou donkeys. Notes: **(A)** the side view of a 17 thoracic vertebrae specimen; **(B)** the side view of an 18 thoracic vertebrae specimen; **(C)** the side view of a 19 thoracic vertebrae specimen; **(D)** the vertical view of 5 lumbar vertebrae specimen; **(E)** the vertical view of 6 lumbar vertebrae specimen. In each figure, the red arrows mark the starting and ending points of the thoracic or lumbar vertebrae, respectively. The blue arrows indicate the straight-line length of the thoracic or lumbar vertebrae. Red numbers with units indicate the straight-line length value of the thoracic or lumbar vertebrae. Red numbers without units indicate the order of the thoracic or lumbar vertebrae. “LN” means the number of lumbar vertebrae; “TN” means the number of thoracic vertebrae.

The multi-vertebrae trait has also attracted donkey breeding researchers since it was discovered several decades ago owing to of multi-vertebrae advantages. Furthermore, the multi-vertebrae trait is highly heritable [heritability is 0.62 in pigs (Borchers et al., 2004)]. For the majority of Dezhou donkeys, there are 18 thoracic vertebrae and 5 lumbar vertebrae, usually labeled as T18L5. To date, we have observed five thoracic-lumbar vertebrae combination types in Dezhou donkeys (T17L5, T17L6, T18L5, T18L6, and T19L5); we have not observed the T19L6 type, nor have we seen any article reporting this. We collected skeletal specimens of three thoracic vertebrae types and two lumbar vertebrae types from the Dezhou donkey slaughterhouse (Figure 1). Many articles have reported that the *LTBP2* gene may have the function of regulating the vertebrae development, but the mechanism is not clear. Zhang et al. (2016) found that growth differentiation factor 11 (*Gdf11*) can increase the number of ribs in mice, while some scholars have found that *LTBP2* can inhibit the extracellular processing of *Gdf11* through basic amino acid specific preprotein convertase 5/6 (*PC5/6*). In our previous studies, through the genome-wide association study (GWAS), we predicted that *LTBP2* may be associated with the vertebrae number in Dezhou donkeys (Wang et al., 2020). Therefore, the current study aims to detect mutations in the *LTBP2* gene and analyze their association with the TLN, body size, and carcass traits of Dezhou donkeys. This study contributes to the understanding of the mechanism based of TLN differences and provides theoretical support for multi-vertebrae molecular marker-assisted selection in donkey breeding.

## 2 Materials and methods

### 2.1 Experimental animals and data acquisition

The study collected 392 Dezhou donkeys in Dezhou Town, Shandong Province, China, from 2019 to 2021 (the average body size data are shown in Supplementary Table S1). All donkeys were 2-year-old jackasses, fattened for meat production, with unknown pedigree information. During this study, the animal feed consisted of grass and hay *ad libitum* and water. The was no food intake within 12 h before slaughter, but water was unlimited. A 10 ml blood sample was collected from each Dezhou donkey using an EDTA anticoagulated blood collection tube and stored at −80°C. The body size [body height (BH), the vertical distance from the highest point of bun nail to the ground; body length (BL), the linear distance from the fore-end of humeral carina to the last internal carina of ischial tubercle; and chest circumference (CHC), make a vertical line from the back of the scapula and measure the circumference of its chest] of these 392 Dezhou donkeys was measured using Zhang et al.’s (2021) method. The measurement for collecting the thoracolumbar vertebrae number and carcass traits were as follows. The warm carcass weight [removing the head, viscus, skin, hooves, penis, testicles, and tail (CW)], the front part of the carcass weight [cut off from the boundary between the last thoracic vertebra and the first lumbar vertebra, and the carcass where the thoracic vertebra are located (CWF)], and the hind part of the carcass weight [cut off from the boundary between the thoracic vertebra and the lumbar vertebra, and the carcass where the lumbar vertebra are located (CWA)] were measured immediately after slaughter. The information regarding thoracolumbar vertebrae was measured [thoracic vertebrae number (TN), the total length of thoracic vertebrae (TL), lumbar vertebrae number (LN), and the total length of lumbar vertebrae (LL)] at the abattoir in the cold-storage room after slaughter on the left half of the carcass (Liu et al., 2022). The TIANAMP Genomic DNA Extraction Kit (DP304, TIANGEN, Beijing, China) was utilized to extract the genomic DNA from blood samples. The A260/280 ratios of all DNA samples were determined with a NanoDrop (ND 2000, NanoDrop, United States). After that, genomic DNA were diluted to a common centration 50 ng/μL and stored at −20°C.

### 2.2 Genotyping

All animals were sequenced for the whole *LTBP2* gene with GBTS (genotyping by targeted sequencing) liquid chip (using GenoBaits and GenoPlexs technology), which was developed by our laboratory and the Shijiazhuang Breeding Biotechnology Co., Ltd. In order to confirm the accuracy of the chip sequencing results, eight pairs of specific primer sequences were designed using Primer Premier 5.0 software to amplify some polymorphic loci of the *LTBP2* gene (50%). The primers are listed in Supplementary Table S2. The fragments contained 16 SNVs (c.5547 + 860 C > T, c.5251 + 281 A > C, c.1381 + 768 T > G, c.1003 + 704 C > T, c.812 + 22526 T > G, c.812 + 6591 T > C, c.565 + 3453 C > T, and c.494 + 5808 C > T) of *LTBP2*. The PCR system (25 μL) included: 2 μL genomic DNA (25 ng/μL); 1 μL of each primer (10 μM); 12.5 μL 2 × MasterMix (TIANGEN, Beijing, China); and 8.5 μL double-distilled H_2_O. The PCR protocol was as follows: 95°C for 10 min; 35 cycles of denaturing at 95°C for 30 s; annealing at Tm°C (Supplementary Table S2) for 30 s; and extension at 72°C for 1 min, with a final extension at 72°C for 10 min. After amplification, PCR products were first electrophoresed on 2% agarose gels with MF079-M5 Hipure Gelred nucleic acid strain (Mei5 Biotechnology, Co., Ltd., Beijing, China) and then sent to Sangon Biotech Biotechnology Co., Ltd. for sequencing in both directions and the sequencing results were compared using DNAMAN software (Version 5.2, Lynnon Biosoft, Vaudreuil, Canada).

### 2.3 Statistical analysis

The genotype frequencies of all target loci (the frequency of each genotype is greater than 5%) were calculated. Using these SNVs, haplotypes were constructed, and haplotype frequency was calculated; haplotypes with frequencies greater than 5% were used for subsequent analysis. The genotype frequencies, allelic frequencies, and Hardy–Weinberg equilibrium partial values were determined by direct counting. At the same time, the population genetic parameters were estimated using online software (http://analysis.bio-x.cn/myAnalysis.php) and (http://www.msrcall.com/Gdicall.aspx), including observed heterozygosity (Obs-Het), predicted heterozygosity (Pred-Het), homozygosity (Ho), effective number of alleles (Ne) (Nei and Roychoudhury, 1974), and polymorphism information content (PIC) (Botstein et al., 1980). The linkage relationship between D’ (LD’ coefficient) and *r* ^2^ (correlation coefficient) based on the alleles of each site was calculated using Haploview software (Version 4.2, Daly Lab at the Broad Institute Cambridge, United States) and haplotype frequency was estimated between the loci (Barrett et al., 2005). In order to explore the differences in SNVs’ genotype and haplotypes in *LTBP2*, which are important genetic factors for several traits, the linear model in SPSS 22.0 software (Statistical Product and Service Solutions, Version 22.0 Edition, IBM, Armonk, NY, United States) was used for correlation analysis:

$$y_{ij}=\mu +a_{i}+e_{ij},$$

where y_ij_ is the measured value of the related trait, μ is the population mean, a_i_ is the genotype effect, and e_ij_ is the random residual.

## 3 Results

### 3.1 Variation in vertebral number in Dezhou donkeys

**FIGURE 2:**
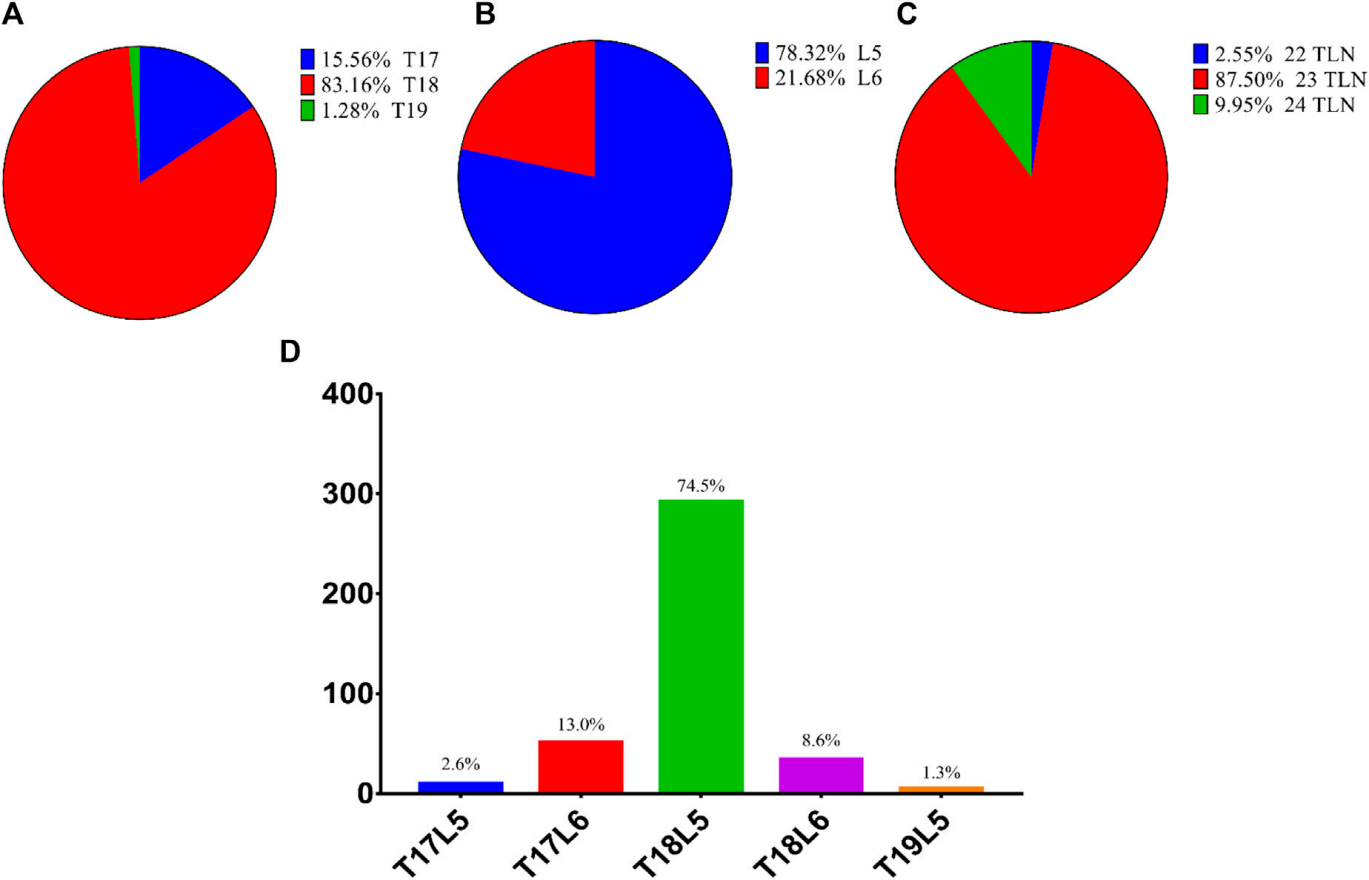
Variation in vertebral number of Dezhou donkeys. Notes: **(A)** variation in TN; **(B)** variation in LN; **(C)** variation in TLN; **(D)** variation in TL-type.

The variation in vertebral number among 392 Dezhou donkeys is shown in Figure 2. The TN, LN, and TLN ranged from 17 to 19, 5 to 6, and 22 to 24, respectively; the dominated type was 18 (83.2%), 5 (78.3%), and 23 (87.5%), respectively. The most common proportion among the experimental samples was the T18L5 thoracic-lumbar vertebrae combinational type (74.5%), and the rarest was T19L5 (1.3%).

### 3.2 Effect of TLN variation on body size and carcass traits

The number of vertebrae is associated with body length, carcass weight, and other economical traits in livestock. However, for Dezhou donkeys, the effect of the vertebrae number increase on economic traits is still unclear. To evaluate their relationship, the effects of single TL, LN, TLN, and thoracic-lumbar vertebrae combinational types on carcass weight were analyzed successfully. In order to accurately calculate the effect of adding one type of vertebrae on body length and carcass weight, we studied the effect of variations in the number of thoracic vertebrae in the same population of lumbar vertebrae and *vice versa* (Supplementary Table S3). When LN remains unchanged, adding a thoracic vertebra means a significant increase in TL by 5 cm according to the weighted average method (TN = 17, TL = 68.73 ± 0.35; TN = 18, TL = 73.61 ± 0.18; TN = 19, TL = 75.2 ± 1.59), significant at *p* < 0.05. When TN remains unchanged, adding a lumbar vertebra means a significant increase in LL by 4 cm according to the weighted average method (LN = 5, LL = 23.27 ± 0.07; LN = 6, LL = 27.19 ± 0.15, *p* < 0.05). From the perspective of TLN, adding a thoracolumbar vertebra means the total length of thoracolumbar vertebrae (TLL) will increase significantly (TLN = 22, TLL = 91.6 ± 1.07; TLN = 23, TLL = 96.75 ± 0.22; TLN = 24, TLL = 100.59 ± 0.71; *p* < 0.05). Individuals with 23 or 24 TLN were about 10 kg heavier than 22 TLN individuals in carcass weight, but no significant difference was detected. Theoretically, there should be a T19L6 combinational type, but this has not been observed in our long-term observations; maybe we need a larger sample size.

### 3.3 Polymorphisms of 16 multi-vertebrae trait causal loci

**FIGURE 3:**
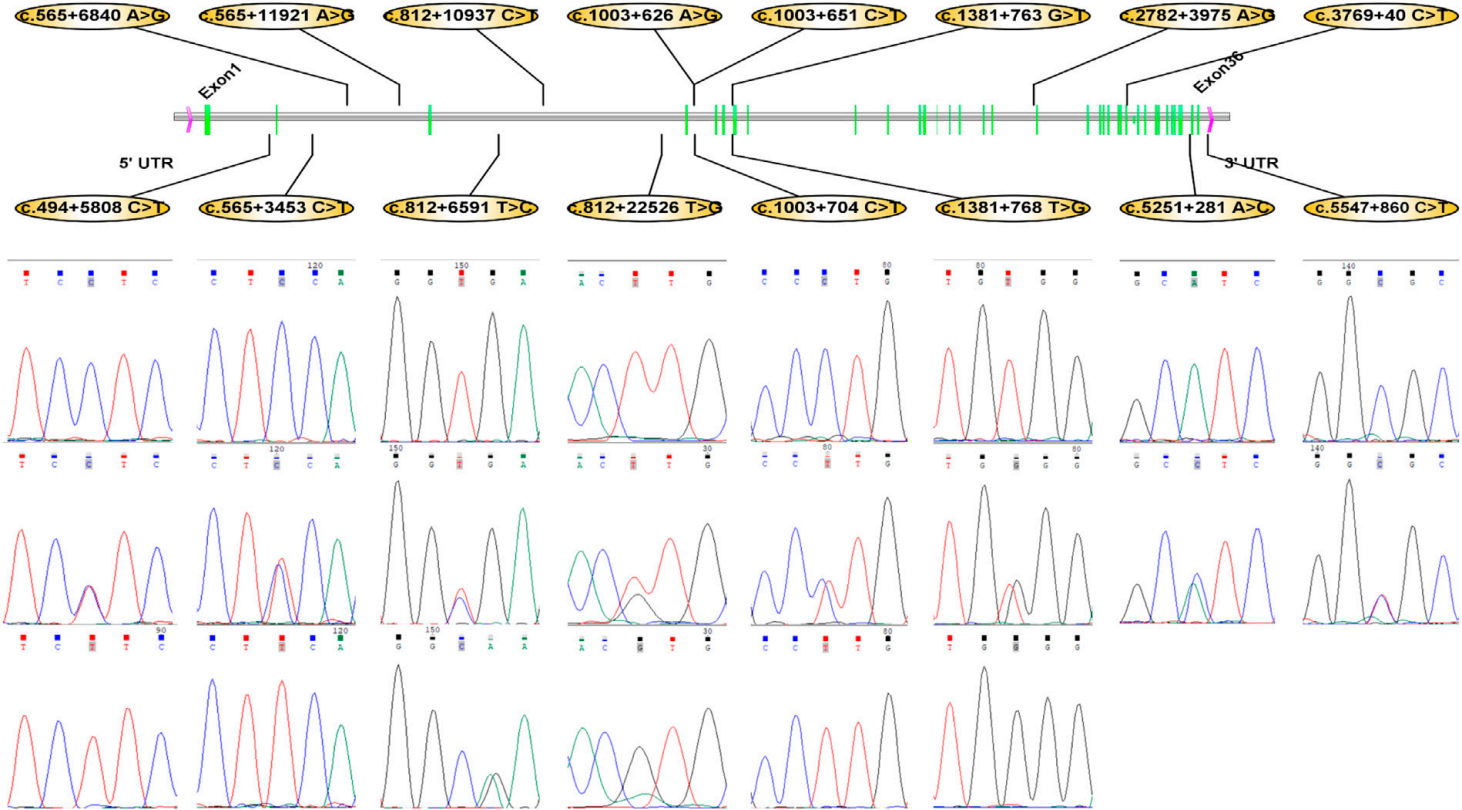
Sixteen SNVs of the *LTBP2* gene in Dezhou donkeys.

A total of 16 mutations (c.5547 + 860 C > T, c.5251 + 281 A > C, c.3769 + 40 C > T, c.2782 + 3975 A > G, c.1381 + 768 T > G, c.1381 + 763 G > T, c.1003 + 704 C > T, c.1003 + 651 C > T, c.1003 + 626 A > G, c.812 + 22526 T > G, c.812 + 10937 C > T, c.812 + 6591 T > C, c.565 + 11921 A > G, c.565 + 6840 A > G, c.565 + 3453 C > T, and c.494 + 5808 C > T) were identified as candidate sites for multi-vertebrae and carcass traits (Figure 3). In order to further confirm their association with vertebrae number, body size, and carcass traits, all samples were genotyped for these 16 alleles using GBTS liquid chip. The sequencing results of the PCR products amplified by specific primers were consistent with those of the GBTS liquid chip.

In Supplementary Table S4, three genotype frequencies (wild-type homozygote, heterozygotes, and mutant-type homozygote) and genetic indices (Obs-Het, Pred-Het, Ho, and PIC) in Dezhou donkeys’ *LTBP2* gene are shown. As shown in Supplementary Table S4, we found that c.5547 + 860 C > T, c.5251 + 281 A > C, c.3769 + 40 C > T, and c.2782 + 3975 A > G did not have a mutant-type homozygote. The other 12 loci had three genotypes in total. The Hardy–Weinberg test results showed that the population was genetically balanced and belonged to the Mendelian population. The genetic diversity index showed that the PIC of Dezhou donkeys was 0.123–0.555, and the polymorphism was 1 < PIC < 0.25, which is in the moderate-high polymorphism state.

### 3.4 Association analysis of donkey vertebrae number, body size, and carcass traits with 16 SNV polymorphisms

The effects of the *LTBP2* SNVs on the number of vertebrae, body size, and carcass traits were evaluated and are presented in Supplementary Table S5. As shown in Supplementary Table S5, individuals with the wild-type homozygote had significantly more TN than heterozygotes (*p* < 0.05) in c.5547 + 860 C > T, c.5251 + 281 A > C, c.3769 + 40 C > T, and c.2782 + 3975 A > G loci, which means the main effect of these four loci are significant in TN. The lumbar vertebrae number in Dezhou donkeys with wild-type homozygous and mutant-type homozygous genotypes of c.1381 + 768 T > G and c.1381 + 763 G > T was 5.05 ± 0.05 and 5.25 ± 0.03, respectively, and the difference was significant (*p* < 0.05). The other two mutant loci significantly related to the number of lumbar vertebrae are c.812 + 10937 C > T and c.812 + 6591 T > C. In c.812 + 10937 C > T, individuals with mutant-type homozygous T/T and heterozygous C/T genotypes had more LN than wild-type homozygotes, while in c.812 + 6591 T > C, individuals with mutant-type homozygous C/C had significantly fewer LN than wild-type homozygotes and heterozygotes. As described in Supplementary Table S5, in the c.1003 + 704 C > T, c.1003 + 651 C > T, c.1003 + 626 A > G, and c.812 + 22526 T > G mutational sites, the effect of variation on CHC, CWF, CWA, and CW was consistent. Individuals with wild-type homozygotes had significantly higher data than other genotypes among the aforementioned four traits (*p* < 0.05), while others (BH, BL, SW, TN, TL, STL, LN, LL, SLL, TLN, TLL, and STLL) were not significant. There are five mutational sites significantly associated with TLN (c.812 + 10937 C > T, c.565 + 11921 A > G, c.565 + 6840 A > G, c.565 + 3453 C > T, and c.494 + 5808 C > T). The effect of c.812 + 10937 C > T in TLN was consistent with that in LN. In c.565 + 11921 A > G, c.565 + 6840 A > G, c.565 + 3453 C > T, and c.494 + 5808 C > T mutational sites, individuals with the heterozygous genotype had the most TLN (23.12 ± 0.03) compared to other genotypes (significantly).

### 3.5 Linkage disequilibrium and haplotype analysis of *LTBP2* gene mutational sites

In order to reveal the linkage relationship between the 16 mutational sites of the *LTBP2* gene, the linkage disequilibrium between these loci was estimated. The analysis results showed that three blocks exist between these 16 SNVs (*r* ^2^ > 0.33) (Ardlie et al., 2002). Block1 is composed of c.5547 + 860 C > T, c.5251 + 281 A > C, c.3769 + 40 C > T, and c.2782 + 3975 A > G, block2 is composed of c.1381 + 768 T > G, c.1381 + 763 G > T, c.1003 + 704 C > T, c.1003 + 651 C > T, c.1003 + 626 A > G, and c.812 + 22526 T > G, and block3 is composed by c.565 + 11921 A > G, c.565 + 6840 A > G, c.565 + 3453 C > T, and c.494 + 5808 C > T. The linkage coefficients for other pairs of SNVs were low (*r* ^2^ < 0.33).

Haplotype analysis was performed for the three blocks, and three haplotypes with a frequency greater than 0.05 were identified and labeled as Hap1 (-CAC​AGT​TTG​GAA​TT-), Hap2 (-CAC​AGT​TTG​GGG​CC-), and Hap3 (-CAC​ATG​CCA​TGG​CC-). The haplotype frequencies of Hap1, Hap2, and Hap3 in Dezhou donkeys account for 37.6%, 28.3%, and 10.2%, respectively.

### 3.6 Association analysis between the haplotype combinations and TLN, body size, and carcass traits

In order to explore the association between SNV polymorphism and the traits of Dezhou donkeys, haplotype combination was carried out on the basis of constructed haplotypes. Six haplotype combinations (Hap1Hap1, Hap1Hap2, Hap1Hap3, Hap2Hap2, Hap2Hap3, and Hap3Hap3) were constructed, respectively. The results are shown in Supplementary Table S6; individuals with Hap2Hap3 and Hap3Hap3 made up less than 5% of the total sample size, so no follow-up analysis was conducted for these two haplotype combinations. The correlation analysis between four haplotypes and TLN, body size, and carcass traits was calculated, but no significant difference was observed.

## 4 Discussion

With the development of agricultural mechanization, the causative function of donkeys is gradually weakening, which is the reason why the stock of donkeys in China decreased rapidly from 1990 to 2016 (Statistics, 2021). In recent years, luckily, with the further study of the special functions of donkey meat, milk, and skin, the situation has gradually improved (D’Auria et al., 2011; Li et al., 2006; Polidori et al., 2015; Polidori et al., 2008; Yvon et al., 2018). Donkeys are raised as a small special livestock in China today; the government has enacted many policies to support the development of the donkey industry, especially in breed preservation and improvement. Vertebrae number is an important characteristic in livestock, such as pigs, cattle, sheep, and donkeys, because a higher vertebrae number means a longer body length and a heavier carcass weight (Yang et al., 2016; Zhang et al., 2017). In this study, we observed that the TN, LN, and TLN in Dezhou donkeys is 17–19, 5–6, and 22–24, respectively; no T19L6 thoracolumbar vertebrae combination type individuals were observed, which is similar to what has been reported in previous studies (Jamdar and Ema, 1982; Gao, 2020; Gao et al., 2021). The effect of increasing TN or LN is not consistent; one more TN increases TLL by about 5.14 cm, while one more LN increases TLL by about 4.05 cm (significant at *p* < 0.05). Interestingly, the effect of one more TN in different LN populations is unequal, such as in the 5 lumber vertebrae number population, the CW of individuals with 18 TN is 11.1 kg more than individuals with 17 TN, while in 6 lumbar vertebrae number population, the CW of individuals with 18 TN is 3 kg more than individuals with 17 TN; the disparity between the increase between the two is 8.1 kg. Similarly, the effect of one more LN in different TN populations is also unequal.

The heritability of the vertebrae is high; for example, the heritability of vertebrae in pigs is 0.62 (Borchers et al., 2004). Therefore, we believe that directional breeding of Dezhou donkeys with multiple thoracolumbar vertebrae numbers and studying the molecular mechanism of multiple thoracolumbar vertebrae numbers is worthy of attention. The development of vertebrae is a complex process, which requires the regulation of multiple signal molecules and specific components. To date, there are several regions in the genome that have been identified as having the function of regulating the development of vertebrae. Mikawa et al. (2005), Mikawa et al. (2007), and Mikawa et al. (2011) identified two QTLs’ influence on the number of vertebrae, mapped to SSC1 and SSC7. There is a 479 kb region on SSC7 including nine annotated genes, identified as a critical fragment influencing the TLN (Liu et al., 2020). The Notch receptor 1 (Notch-1) gene is a critical gene for somite development, while the activation of FOS could inhibit the expression of Notch-1 (Portanova et al., 2013; Liao and Oates, 2017).

In previous studies, many mutation sites in *LTBP2* have been proved to have some functions in the development of primary congenital glaucoma (c.3028G > A, p.Asp1010Asn; c.3427delC, p.Gln1143Argfs*35) (Rauf et al., 2020), cardiomyocytes (c.2206G > A, p.Asp736Asn) (Chen et al., 2020), and lung fibroblast-to-myofibroblast differentiation (Zou et al., 2021). *LTBP2* has also been found to be related to the number of teats in pigs (Martins et al., 2022). In this study, *LTBP2* was used as a candidate gene associated with TLN in Dezhou donkeys based on related studies (Zhao et al., 2020; Wang et al., 2022). The liquid chip was designed based on published data from several databases and was used for genotyping all mutational loci of all samples. PCR primers were designed, based on a published donkey *LTBP2* sequence (GenBank accession NC-052183.1), to amplify the eight mutational loci (Wang et al., 2020). The alignments of the genotyping result between GBTS liquid chip and PCR products with specific primers is 100%. From this, we can see that the GBTS liquid chip sequencing results are reliable.

We actually detected a total of 544 mutational sites in the whole *LTBP2* gene of Dezhou donkeys; however, most of the loci cannot meet the requirements of correlation analysis, so we finally screened out 16 SNVs for subsequent analysis. Among these loci, the adjacent loci are significantly related to a certain trait (*p* < 0.05), centrally. For example, for c.5547 + 860 C > T, c.5251 + 281 A > C, c.3769 + 40 C > T, and c.2782 + 3975 A > G, the distance between them was 8,247 bp, 6,018 bp, and 9,253 bp, respectively, genome wide; they were all significantly associated with TN (*p* < 0.05). The analysis results from the Haploview software showed a strong linkage disequilibrium between them, which means when the C allele is mutated to the T allele at c.5547 + 860 C > T, the A allele at c.5251 + 281 A > C will mutate to C allele, the C allele at c.3769 + 40 C > T will mutate to T allele, and the A allele at c.2782 + 3975 A > G will mutate to G allele. In this block, individuals with wild-type homozygous had more 0.16 pieces of TN than heterozygotes, so we speculate that the mutation here has a negative effect on TN. The same situation also occurs in block2 (c.1381 + 768 T > G, c.1381 + 763 G > T, c.1003 + 704 C > T, c.1003 + 651 C > T, c.1003 + 626 A > G, and c.812 + 22526 T > G), but the function of block2 is aimed at LN, CHC, CWF, CWA, and CW. For the development of TN, mutations at c.1381 + 768 T > G and c.1381 + 763 G > T sites are beneficial, while for CHC, CWF, CWA, and CW, mutations at c.1003 + 704 C > T, c.1003 + 651 C > T, c.1003 + 626 A > G, and c.812 + 22526 T > G are negative. In block3, four SNVs (c.565 + 11921 A > G, c.565 + 6840 A > G, c.565 + 3453 C > T, and c.494 + 5808 C > T) were all significantly associated with TLN; individuals with the heterozygous genotype had higher TLN than others (*p* < 0.05), but the difference between wild-type homozygotes and mutant-type homozygotes was different.

In order to determine the linkage disequilibrium of 16 SNV markers located on the Dezhou donkey *LTBP2* gene, a total of three haplotype blocks among 16 SNVs were identified, examining haplotypes above 5%, spanning approximately 30 kb. The most frequent haplotypes within the blocks comprised the linkage of nucleotides CACA (96.4%) for block1, GTTTGG (72.8%) for block2, and AATT (53.6%) for block3. To demonstrate the total effect of a single locus, haplotype combinations were combined for further study. The haplotype combination types with a sample size less than 5% of the total sample were removed. Our results showed no dominant type in the four haplotype combinations; the reason may be the effect of these three blocks counteracting each other. These results indicate that the *LTBP2* gene is a potential functional gene for regulating the development of vertebrae in Dezhou donkeys, and this finding may provide important implications for further studies on the regulation of the TLN in Dezhou donkeys. It must be noted that our research has not explored the molecular mechanism of SNPs in *LTBP2* regarding how they effect TLN development.

## 5 Conclusion

In summary, the effects of TLN variation on body size and carcass traits of Dezhou donkeys were quantified, and 16 trait-related SNVs have been identified in the *LTBP2* gene of Dezhou donkeys. These loci have low-to-high polymorphism and there are significant differences between different genotypes among different traits. The results of this study will play an important role in the further study of the molecular mechanism of Dezhou donkey molecular breeding for muti-vertebrae breeds.

## Data Availability

The datasets presented in this study can be found in online repositories. The name of the repository and accession number can be found at: NCBI; PRJNA878942.
