# Supplementary material for: Identification of LTBP2 gene polymorphisms and their association with thoracolumbar vertebrae number, body size, and carcass traits in Dezhou donkeys
Source: Front Genet. 2022 Nov 22;13:969959. doi: 10.3389/fgene.2022.969959 (PMC9723334; doi:10.3389/fgene.2022.969959)
Supplement: Supplementary file 1 [file Table2.DOCX]

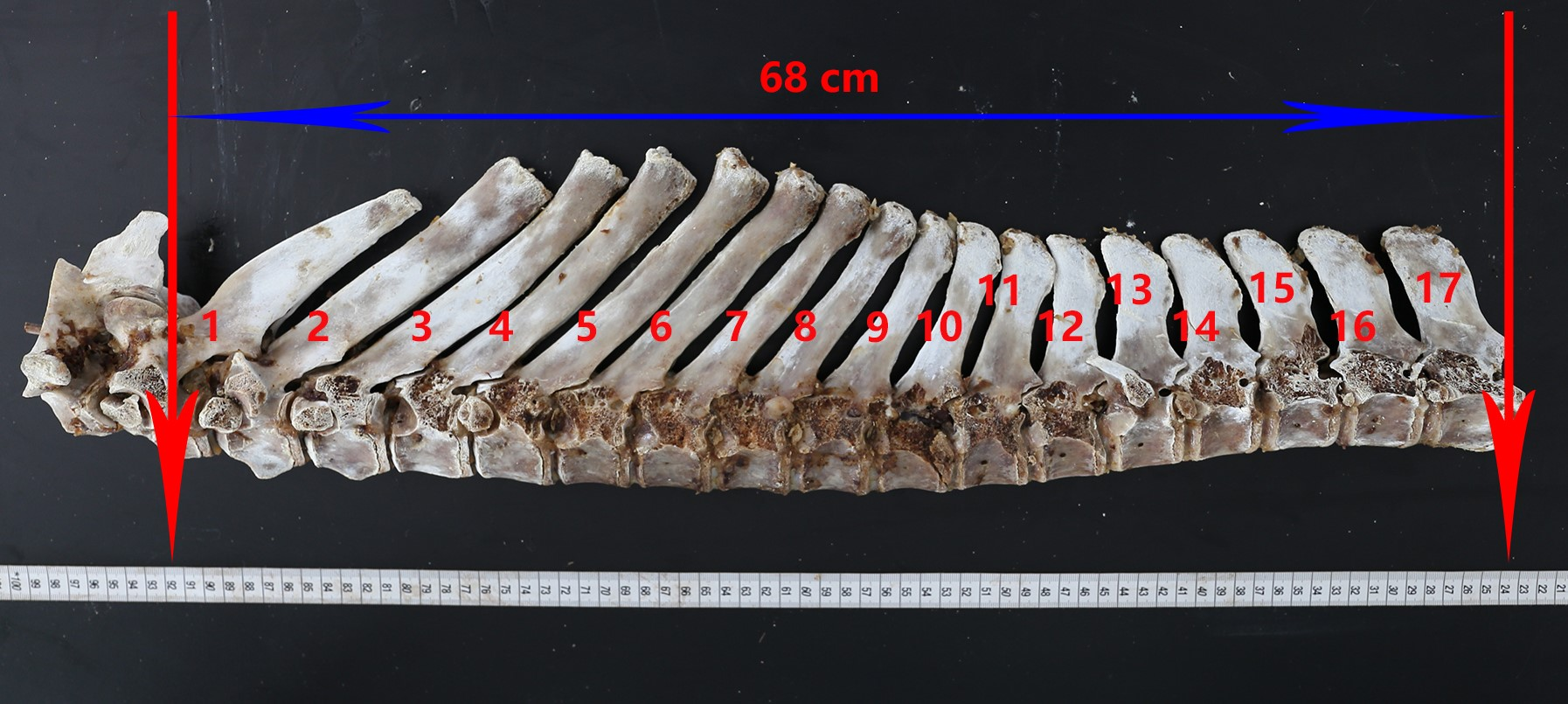


**TN=17 (side view)**

**A**


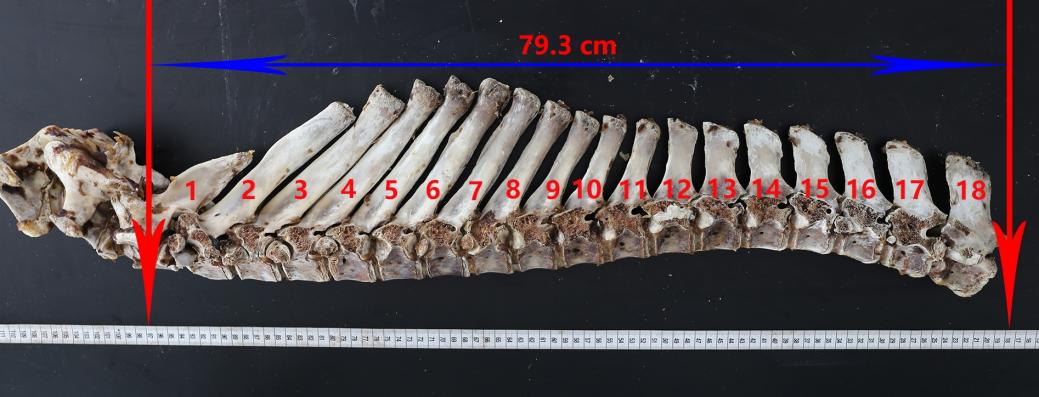


**TN=18 (side view)**

**B**


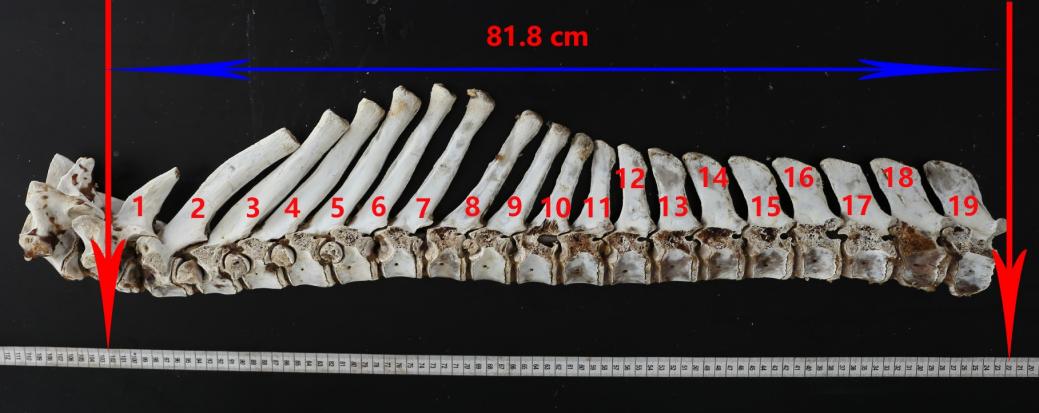


**TN=19 (side view)**

**C**

**E**

**D**


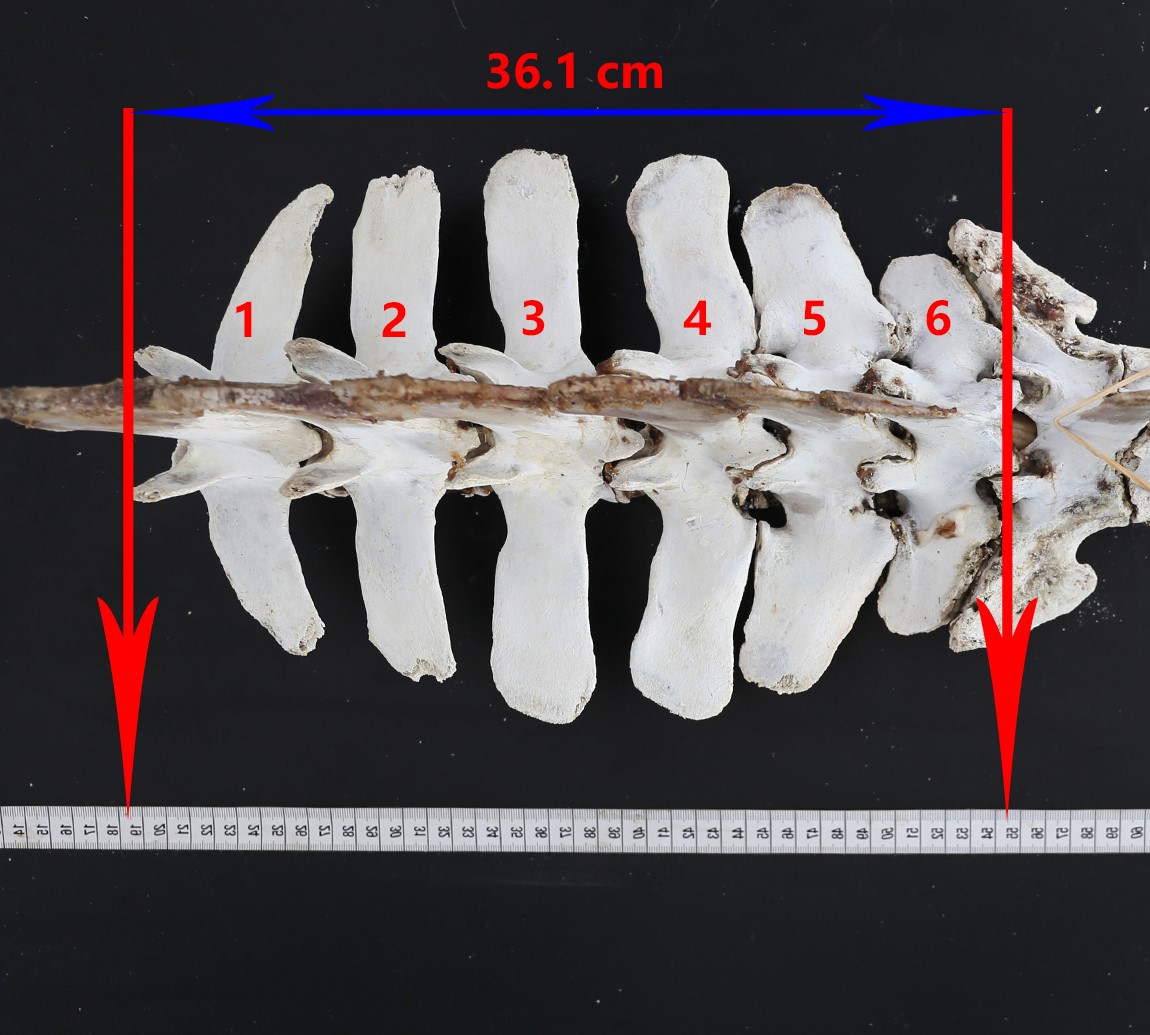

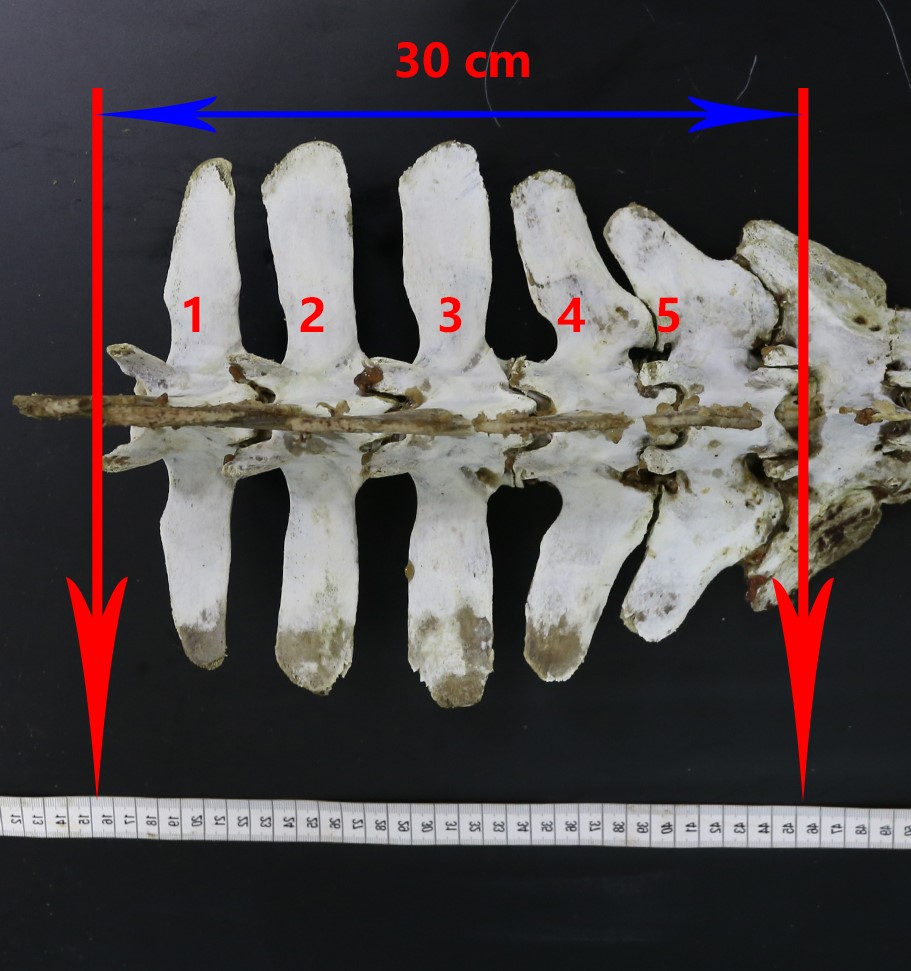


**LN=5 (vertical view)**

**LN=6 (vertical view)**

Figure 1. Thoracic and lumbar vertebrae specimens of Dezhou donkey

Note: (A) The side view of 17 thoracic vertebrae specimen, (B) The side view of 18 thoracic vertebrae specimen, (C) The side view of 19 thoracic vertebrae specimen, (D) The vertical view of 5 lumbar vertebrae specimen, (E) The vertical view of 6 lumbar vertebrae specimen.

In each figure, the red arrows mark the starting and ending points of thoracic or lumbar vertebrae, respectively. The blue arrow indicates the straight-line length of the thoracic or lumbar vertebrae. The red number with units indicates the straight-line length value of the thoracic or lumbar vertebrae. The red number without unit indicates the order of the thoracic or lumbar vertebrae. “LN” means the number of lumbar vertebrae, “TN” means the number of thoracic vertebrae.


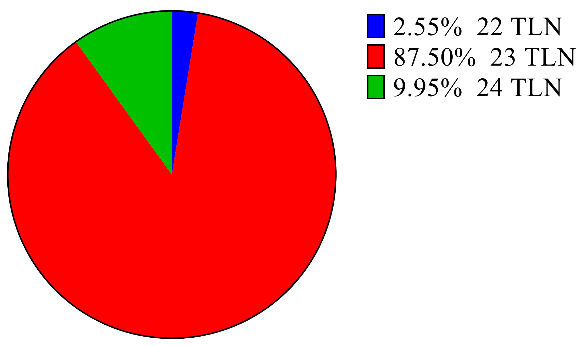

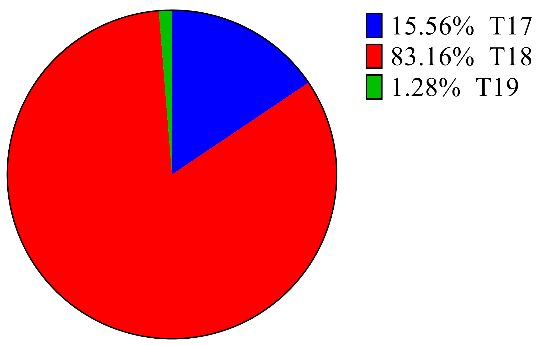

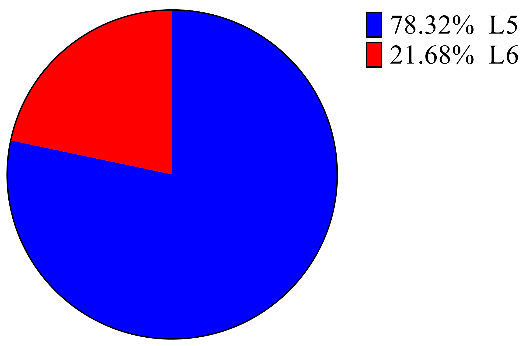


A

B

C


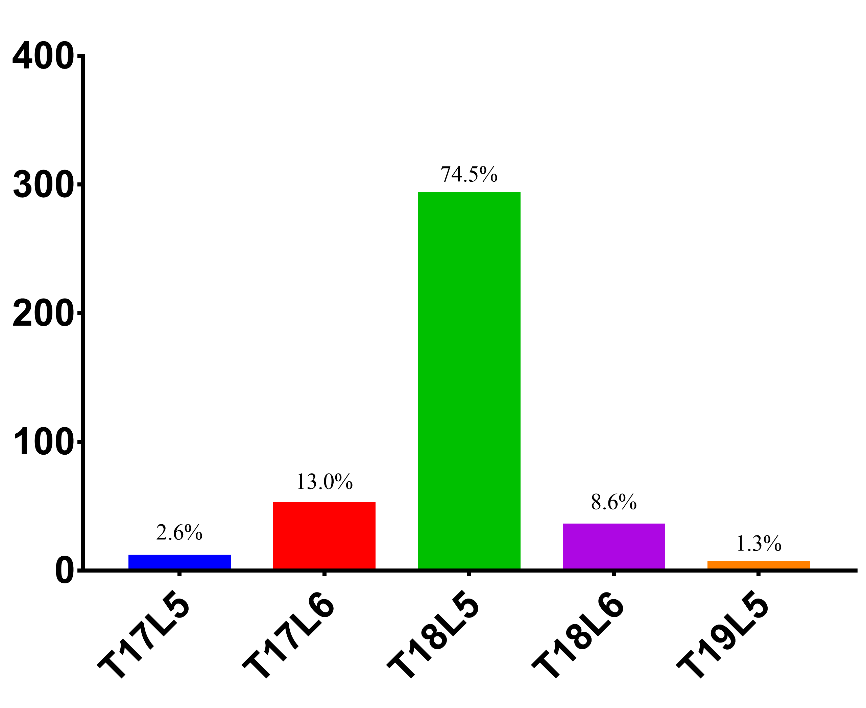


D

Figure 2. The variation of vertebral number of Dezhou donkeys

Note: (A) The variation of TN in Dezhou donkeys, (B) The variation of LN in Dezhou donkeys, (C) The variation of TLN in Dezhou donkeys, (D) The variation of TL-Type in Dezhou donkeys.


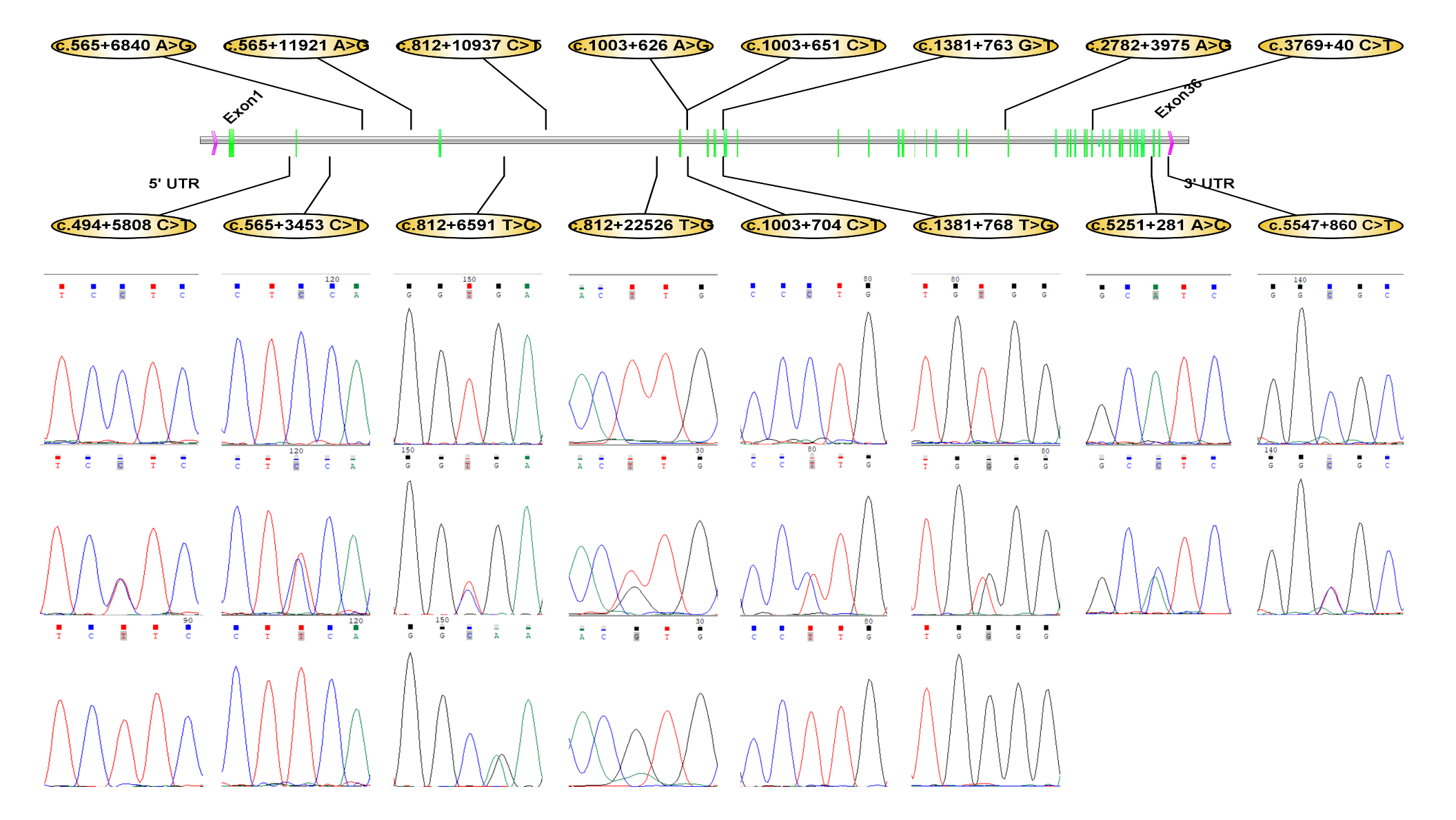
Figure 3. 16 SNVs of *LTBP2* gene in Dezhou donkeys
